# A rare concomitance of Wilson’s disease and systemic lupus erythematosus in a teenage girl: a case report and literature review

**DOI:** 10.3389/fped.2023.1296426

**Published:** 2024-01-08

**Authors:** Zigui Yang, Yashuang Su, Meilu Liu, Lijun Sun, Fengxiao Zhang, Wei Lin

**Affiliations:** ^1^ Department of Rheumatology and Immunology, Hebei General Hospital, Shijiazhuang, Hebei, China; ^2^ Department of Graduate School, Hebei Medical University, Shijiazhuang, Hebei, China

**Keywords:** Wilson's disease, systemic lupus erythematosus, liver cirrhosis, diagnosis, case report

## Abstract

**Background:**

Wilson's disease (WD) is an inherited disorder characterized by impaired biliary excretion of copper and excessive copper accumulation in multiple organs, primarily leading to hepatic, neurological, and psychiatric manifestations. The coexistence of WD and systemic lupus erythematosus (SLE) has rarely been reported, posing challenges in accurately diagnosing these two conditions because of overlapping clinical symptoms.

**Case presentation:**

We presented the case of a 17-year-old girl initially suspected of having SLE due to positive anti-nuclear antibodies and lupus anticoagulants, decreased platelet count, hypocomplementemia, and pleural effusion. However, the patient also exhibited an unusual manifestation of decompensated liver cirrhosis, which is not typical of SLE. Further investigation revealed low serum ceruloplasmin levels, high 24-h urine copper levels, the presence of Kayser–Fleischer rings, and a compound heterozygous mutation in the *ATP7B* gene, confirming the diagnosis of WD.

**Conclusion:**

The co-occurrence of WD and SLE poses a significant diagnostic challenge, often leading to misdiagnosis and delayed diagnosis. Therefore, in patients with well-controlled SLE presenting with unexplained liver fibrosis, neurological involvement, or psychiatric symptoms, it is crucial to consider the possibility of WD. However, further studies are required to elucidate the underlying pathophysiological mechanisms.

## 1 Introduction

Wilson's disease (WD), or hepatolenticular degeneration (HLD), is a rare autosomal recessive disorder caused by mutations in the *ATP7B* gene on chromosome 13. This genetic alteration leads to impaired biliary excretion of copper and the subsequent accumulation of excess copper in various organs, particularly the liver, brain, and cornea (1, 2). The onset of WD typically occurs between the ages of 5 and 35 years; however, it can also affect individuals under 5 years and over 70 years of age (2, 3). While it is recognized that WD can involve multiple systems, hepatic manifestations, neurological dysfunction, and psychiatric features remain the most prevalent symptoms (2).

Systemic lupus erythematosus (SLE) is an autoimmune disease characterized by the production of various autoantibodies and immune complex-mediated tissue damage in multiple organs, including the skin, kidneys, joints, lungs, heart, digestive system, vessels, and nervous system (4). The co-occurrence of WD and SLE without a history of d-penicillamine treatment has rarely been reported in recent years. In this case report, we present a unique occurrence of liver cirrhosis in a 17-year-old young girl with both WD and SLE to raise awareness of their potential coexistence.

## 2 Case description

In November 2022, a 17-year-old girl was admitted to our hospital complaining of facial and limb edema persisting for 2 months and abdominal distension for the last 20 days, which had worsened in the preceding 2 days. On physical examination, notable finding included facial and limb edema, the presence of ascites, and splenomegaly. She did not exhibit any oral ulcers, rash, arthralgia, fever, jaundice, or neurological symptoms. Laboratory investigations revealed a hemoglobin level of 80 g/L and a platelet count of 100 × 10^9^ /L. During hospitalization, the recorded lowest platelet count was 78 × 10^9^ /L. Urinalysis showed 35% dysmorphic red blood cells (RBCs). The anti-nuclear antibody (ANA) titer was 1:320 (nuclear-speckled pattern). Tests for antibodies against Sjögren's syndrome A antigen (SSA), lupus anticoagulant (LA), and extra-criteria antiphospholipid antibodies, including anti-domain I *β*2GPI antibodies, anti-vimentin antibodies, and anti-annexin A2 antibodies, were positive. The anti-double-stranded DNA antibody (anti-dsDNA) test was negative. Lower levels of complement 3 (C3, 0.290 g/L) and complement 4 (C4, 0.065 g/L) and higher levels of immunoglobulin G (IgG, 17.9 g/L) and immunoglobulin M (IgM, 3.65 g/L) were noted. The patient's liver function test demonstrated elevated levels of aspartate aminotransferase (AST, 57.6 U/L) and gamma-glutamyltransferase (GGT, 78.8 U/L) and lower levels of serum albumin (24.2 g/L) (Table 1). The results of Coombs test (direct and indirect) were negative, but bone marrow analysis showed iron deficiency and mild defective megakaryocyte maturation. Abdominal ultrasound revealed the presence of ascites. Computed tomography (CT) of the chest showed right pleural effusion. According to the 2019 EULAR/ACR classification criteria, eligibility criteria for establishing the diagnosis of SLE are ANA ≥ 1:80, at least one clinical classification standard, and a total score ≥10 points (5). The patient's ANA titer was 1:320, and the total score was 15 points (decreased platelet count, Score 4; positive LA, Score 2; hypocomplementemia, Score 4; pleural effusion, Score 5). Thus, a diagnosis of probable SLE with antiphospholipid antibody syndrome (APLA) was established. The patient started receiving methylprednisolone and hydroxychloroquine (HCQ) treatment.

**Table 1:** Serum results before and after treatment of our patient with concurrent Wilson's disease and SLE.

	First day of admission	1 month	3 months	6 months
White blood cells (×10^9^ /L) (3.5–9.5)	4.68	6.93	9.25	6.52
Red blood cells (×10^12^ /L) (3.8–5.1)	2.7	4.23	4.63	4.62
Hemoglobin (g/L) (115–150)	80	108	108	107
Platelets (×10^9^ /L) (125–350)	100	85	118	149
Total protein (g/L) (65–85)	58.1	61.6	67.8	70.4
Albumin (g/L) (40–55)	24.2	27.5	36.6	41.7
Prealbumin (mg/dl) (18–35)	10.2	9.8	14.5	16.5
Alanine aminotransferase (U/L) (7–40)	16.1	33	31.9	16
Aspartate transaminase (U/L) (13–35)	57.6	34.8	24.6	23.8
Gamma-glutamyltransferase (U/L) (7–45)	78.8	94.4	35.4	23
Total bilirubin (μmol/L) (0–23)	37.4	23.1	27.1	17.4
Conjugated bilirubin (μmol/L) (0–4)	12.3	5.9	5.3	2.9
Prothrombin time (s) (9.8–12.1)	19.6	15.9	13.8	12.2
International standardized ratio of prothrombin (0.85–1.3)	1.78	1.42	1.22	1.05
Activated partial thromboplastin time (s) (23.3–32.5)	34.2	30.5	27.6	27.3
Fibrinogen (g/L) (2–4)	1.24	1.45	2.39	2.35
Ceruloplasmin (mg/dl) (16–45)	5.3	NA	NA	NA
24-h urine copper (μg) (15–60)	460	NA	NA	NA
IgG (g/L) (8.6–17.4)	17.9	18.93	11.52	11.33
IgA (g/L) (1.0–4.2)	3.25	4.24	3.34	2.12
IgM (g/L) (0.5–2.8)	3.65	3.34	3.60	2.63
C3 (g/L) (0.7–1.4)	0.29	0.38	0.73	0.92
C4 (g/L) (0.1–0.4)	0.065	0.069	0.155	0.136
ESR (mm/h) (0–20)	10	10	8	6
CRP (mg/L) (0–6)	3.38	7.49	1.35	0.24
24-h urinary protein (g) (0.028–0.141)	0.14	NA	NA	NA
ANA (−)	1:320 (+)	NA	NA	NA
Anti-dsDNA Ab (−)	—	NA	NA	NA
Coombs test (−)	—	NA	NA	NA

ESR, erythrocyte sedimentation rate; CRP, C-reactive protein; Ab, antibody; NA, no data available.

**Figure 1:**
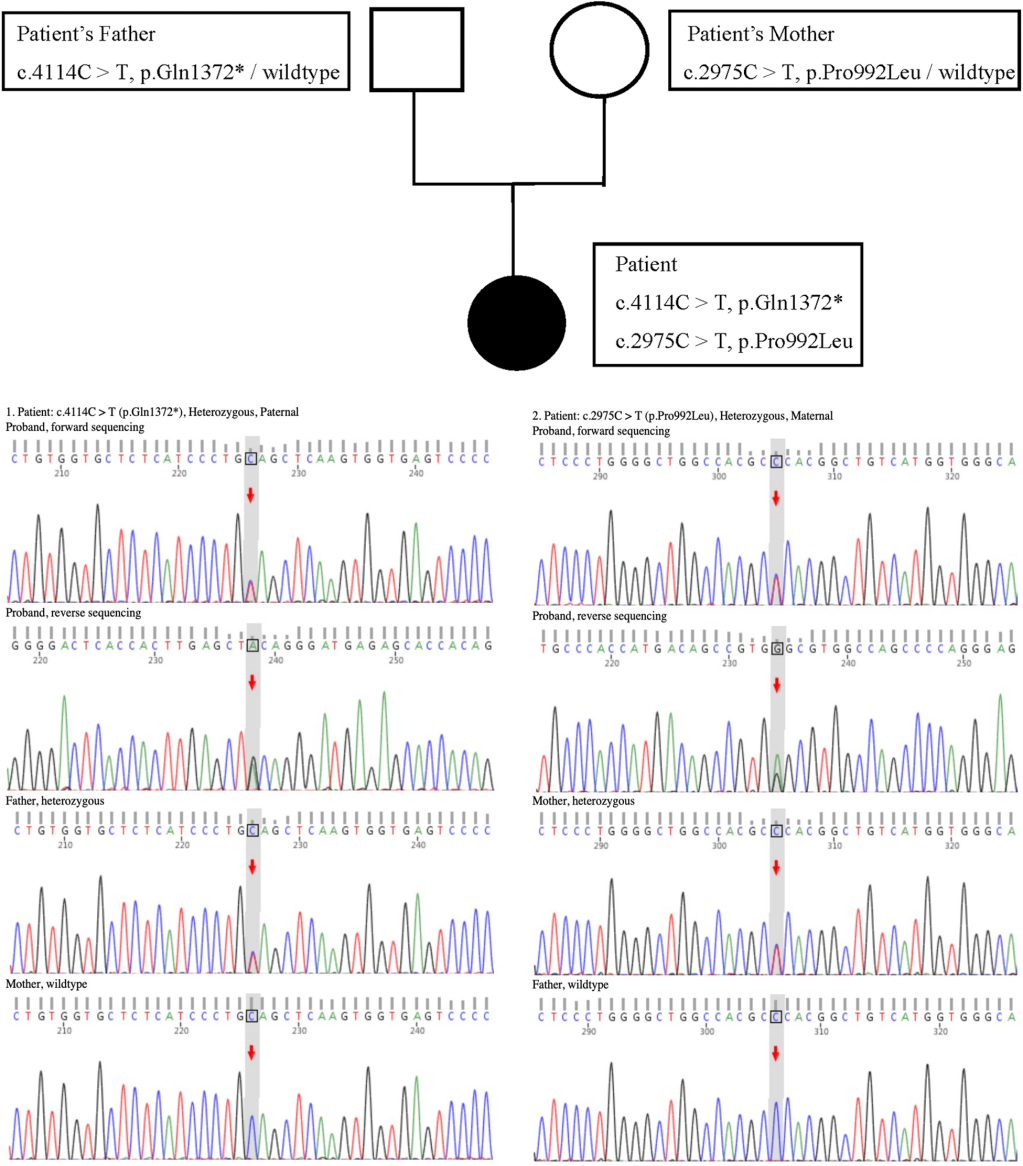
Sequencing results for the *ATP7B* gene in the family.

However, in the absence of proteinuria, the persistent hypoalbuminemia in the patient remained unexplained (the 24-h urinary protein test yielded negative results). Moreover, prothrombin time, the International Standardized Ratio of Prothrombin, and activated partial thromboplastin time were prolonged, and the fibrinogen content was extremely reduced. CT of the upper abdomen showed a diminutive liver volume, irregular edges, uneven hyperechoic nodules in the liver parenchyma and abdominal and pelvic effusion, indicating liver cirrhosis with splenomegaly. A non-invasive diagnosis of liver fibrosis by ultrasound revealed an elevation in liver hardness, which was consistent with Metavir stage > F4. This prompted us to investigate the cause of liver cirrhosis. The patient tested negative for hepatitis B and other hepatitis viruses. Although the ANA test was positive, other autoimmune hepatitis (AIH)-related autoantibodies, such as anti-smooth muscle antibodies (ASMAs) and liver kidney microsomal (LKM-1) antibodies, were found to be negative. The test for primary biliary cholangitis (PBC)-related anti-mitochondrial antibodies (AMA) was also found to be negative. The serum ceruloplasmin level was 5.3 mg/dl (normal range: 16–45 mg/dl), and the 24-h urine copper level was 460 μg/24 h (normal range: 15–60 μg/24 h). A slit-lamp ophthalmological examination confirmed the presence of Kayser–Fleischer (K–F) rings in both eyes. The genotype test for WD revealed that the patient (proband) exhibited a compound heterozygous mutation in the *ATP7B* gene (Figure 1). Both the paternal heterozygous mutation (c.4114C>T, p.Gln1372*) and the maternal heterozygous mutation (c.2975C>T, p.Pro992Leu) were previously confirmed as pathogenic. The Ferenci score for the patient was greater than 4, confirming the WD diagnosis. A liver biopsy aimed at histologic examination and quantification of hepatic copper deposition can aid in the diagnosis of WD and rule out other competing liver diseases. A liver biopsy is only required when clinical signs and non-invasive examinations fail to establish a final diagnosis or when there is suspicion of other liver diseases. Our patient was ultimately diagnosed with WD, and a liver biopsy was not needed.

Based on these findings, the final diagnosis was WD with probable SLE and APLA. Although oral d-penicillamine remains the principal treatment for WD, lupus induced by d-penicillamine is well documented. Considering the co-occurrence of WD and SLE in our case, d-penicillamine is not suitable for WD treatment. Therefore, the patient was started on oral zinc gluconate tablets (50 mg p.o. t.i.d.) for WD and methylprednisolone (40 mg i.v. q.d. for 16 days, then 36 mg p.o. q.d. for 2 weeks), mycophenolate mofetil (MMF, 0.75 g p.o. b.i.d.), and HCQ (0.2 g p.o. b.i.d.) for SLE. During the subsequent 6-month follow-up, almost all clinical symptoms of the patient, including facial and limb edema and abdominal distension, were resolved. In addition, laboratory tests showed significant improvement. The RBC count; hemoglobin level; platelet count; coagulation function, including prothrombin time, activated partial thromboplastin time, and fibrinogen content; liver function tests, including transaminase, bilirubin, and serum albumin levels; and IgG, IgM, C3, and C4 levels returned to normal ranges (Table 1). The steroid dosage was gradually tapered to 8 mg/day while keeping the therapeutic doses of zinc gluconate tablets, MMF, and HCQ unchanged.

## 3 Discussion

In this case report, we discuss the case of a patient who initially presented with unexplained facial and limb edema, abdominal distension, positive ANA and LA titer, decreased platelet count, hypocomplementemia, and pleural effusion. SLE diagnosis was considered, and the effectiveness of steroids and immunosuppressants also confirmed the diagnosis. However, hypoalbuminemia and coagulation disorders did not improve after steroid therapy. In addition, our patient had an uncommon occurrence of unidentified liver cirrhosis in SLE. SLE can affect multiple organs, including the liver (6). Hepatic dysfunction in SLE can be classified as primary (SLE-related), such as lupus hepatitis, or secondary (non-SLE-related), such as AIH, viral hepatitis, drug-induced liver injury, or non-alcoholic fatty liver disease (7). However, given the patient's negative medical history and hepatitis virus test results, coupled with the exclusion of AIH according to the comprehensive diagnostic scoring system recommended by the International AIH Group (8), this case prompted us to explore other potential causes of liver injury and cirrhosis in a young individual. Ultimately, the diagnosis of WD was confirmed based on low serum ceruloplasmin levels, high 24-h urine copper levels, the presence of K–F rings, and the identification of a compound heterozygous mutation in the *ATP7B* gene.

WD is an inherited monogenic disorder characterized by significant copper accumulation in various tissues and organs due to mutations in the *ATP7B* gene, leading to a wide range of clinical manifestations (9). Liver dysfunction, including acute or chronic hepatitis, acute liver failure, cirrhosis, hepatic encephalopathy, fulminant hepatitis, and asymptomatic liver biochemical abnormalities (10, 11), is one of the most common symptom associated with WD. Similarly, SLE can also cause abnormal liver damage. Furthermore, although rare, the presence of several autoantibodies in WD has been reported, including ANA, ASMA, KLM-1, and even anti-dsDNA antibodies (12–15). Therefore, the coexistence of WD and SLE poses a significant diagnostic challenge that may lead to missed or delayed diagnoses. Correct and timely diagnosis is essential for the treatment of WD and SLE. In our case, the patient was given zinc only for WD because d-penicillamine is not recommended in the context of WD coexisting with SLE. Zinc is effective and well-tolerated in maintenance therapy. However, zinc may not always be effective for hepatic WD in the long term. Hence, the patient should be simultaneously evaluated for liver transplantation, and if the patient does not respond to medical therapy, hepatic transplantation should be considered. The New Wilson Index (NWI), a prognostic scoring system that includes measures of hepatic damage and inflammatory response, may help identify whether the patient's medical treatment is successful, particularly when applied serially (16, 17).

Rare occurrences of concomitant WD and SLE have been documented, including the case presented in this study. In addition to our findings, five previous cases have described simultaneous diagnoses of WD and SLE, with one involving a male patient. Santhakumar et al. reported the diagnosis of SLE in a 24-year-old woman based on classical features and laboratory results. During ophthalmic examinations for visual impairment, K–F rings were incidentally discovered. Further investigations revealed low serum ceruloplasmin levels and high 24-h urine copper levels, confirming the diagnosis of WD (18). Pradhan et al. described a 12-year-old girl from India who presented with recurrent hematuria, deranged coagulation, and elevated AST levels. Investigation of the causes of liver damage revealed decreased serum ceruloplasmin levels, elevated 24-h urinary copper levels, and the presence of K–F rings. A liver biopsy confirmed cirrhosis, leading to a simultaneous diagnosis of WD and SLE (14). In a report by Zhang et al., a young female patient initially presented with typical neuropsychiatric symptoms and was ultimately diagnosed with both WD and SLE (19). A Chinese woman was diagnosed with WD based on the presence of cirrhosis, K–F rings, decreased serum ceruloplasmin levels, elevated 24-h urinary copper excretion, and a mutation in the *ATP7B* gene. In addition, the presence of immune thrombocytopenia (ITP) and multiple positive autoantibodies confirmed the coexistence of SLE (20). Hadef et al. reported the case of a male patient with a definitive diagnosis of both WD and SLE. The patient exhibited unexplained impaired liver function and hemolytic anemia, leading to a diagnosis of WD based on copper tests. However, considering the concomitant nephrotic syndrome and multiple positive autoantibodies present in this case, SLE was also considered a contributing factor (21).

Although penicillamine-induced lupus has been extensively documented in the literature, the etiological association of concomitant WD and SLE has not been established. One hypothesis proposes that the activation of the Fas-ligand pathway-mediated apoptosis may play a role in both diseases. In such patients, caution should be exercised when considering penicillamine treatment.

In conclusion, although our case of concurrent WD and SLE not induced by penicillamine is not the first report, given its rarity, each new case is of significant importance. Furthermore, the current study expands on the simultaneous clinical manifestations associated with WD and SLE. Clinicians should consider WD as a potential diagnosis in patients with well-controlled SLE who present with unexplained liver fibrosis, neurological involvement, or psychiatric manifestations. A timely diagnosis and the implementation of appropriate treatment could improve the prognosis of patients.

## Data Availability

The original contributions presented in the study are included in the article/Supplementary Material, further inquiries can be directed to the corresponding author.
